# Hyperinsulinemia Can Cause Kidney Disease in the IGT Stage of OLETF Rats via the INS/IRS-1/PI3-K/Akt Signaling Pathway

**DOI:** 10.1155/2019/4709715

**Published:** 2019-10-13

**Authors:** Yi Zhang, Shaohua Yang, Xiao Cui, Juhong Yang, Miaoyan Zheng, Junya Jia, Fei Han, Xiaoyun Yang, Jingyu Wang, Zhenhong Guo, Bai Chang, Baocheng Chang

**Affiliations:** ^1^Department of Nephropathy, NHC Key Laboratory of Hormones and Development (Tianjin Medical University), Tianjin Key Laboratory of Metabolic Diseases, Tianjin Medical University Chu Hsien-I Memorial Hospital & Tianjin Institute of Endocrinology, Tianjin 300134, China; ^2^Department of Nephropathy, Tianjin Medical University General Hospital, Tianjin 300052, China; ^3^Department of Endocrine Metabolism, Tianjin Medical University General Hospital, Tianjin 300052, China; ^4^Department of Endocrine Metabolism, Zhengzhou Yihe Hospital, Zhengzhou 450047, China

## Abstract

**Aims:**

We investigated the changes of renal structure and its function in normal glucose tolerance (NGT), impaired glucose tolerance (IGT), diabetes mellitus (DM), and diabetic kidney disease (DKD) stages in OLETF rats and explored the role of the INS/IRS-1/PI3-K/Akt signaling pathway.

**Methods:**

OLETF rats were assigned into four groups on the basis of OGTT results and 24 h urinary microalbumin: NGT, IGT, DM, and DKD groups. The changes of renal structure and function and the corresponding pathological changes were observed. The absorption of albumin and the expression of megalin, cubilin, IRS-1, PI3-K, and Akt in NRK-52E cells were measured after being stimulated by different concentrations of insulin.

**Results:**

In the IGT group, the index which reflects the function of renal tubule-like N-acetyl-*β*-glucosaminidase, neutrophil gelatinase-associated lipocalin, retinol-binding protein, and cystatin C was higher than those in the control group and the NGT group (*P* < 0.05). Significant renal structure damages, especially in renal tubules, were observed in the IGT group. In the presence of insulin at a high concentration, the IRS-1/PI3-K/Akt signaling pathway in renal tubular epithelial cells was inhibited, and the expression of megalin and cubilin was significantly downregulated which was accompanied by a minimum uptake of albumin.

**Conclusions:**

In contrast to DKD, the renal structural damage and functional changes in the IGT stage, in which we propose the term “IGT kidney disease,” mainly manifest as renal tubular injury. Insulin resistance and compensatory hyperinsulinemia may be involved in its pathogenesis.

## 1. Introduction

With a rapid increase in the morbidity rate of diabetes mellitus (DM), diabetic kidney disease (DKD) has become the leading cause of end-stage renal disease (ESRD) [1]. Therefore, the early prevention and treatment of DKD are of great importance. Microalbuminuria (MAU) is the main clinical criterion for the early diagnosis of DKD. However, even after active treatment, only 20%-25% patients will obtain normal urine protein levels within 10 years, 30%-35% of them will still manifest with MAU, and 30%-40% will gradually develop into macroalbuminuria and even ESRD [2]. Krolewski et al. followed up 79 patients with type 1 diabetes for 12 years and found that MAU was neither sensitive nor specific for the prediction of DKD [3]. We have also found that a series of pathophysiological changes has occurred long before MAU in patients with type 2 diabetes leading to renal hypertrophy, abnormal blood pressure, and renal tubular dysfunction [4].

An epidemiological survey showed that some patients had various degrees of kidney damage before or upon the diagnosis of DM [5, 6]. Even in patients with impaired fasting glucose and impaired glucose tolerance (IGT), 13.1% suffered from MAU [7]. Therefore, it is important to further dynamically study the alteration of kidney structure and function during the onset and progression of type 2 DM in different phases: normal glucose tolerance (NGT), IGT, DM, and DKD.

In addition, the pathogenesis of DKD has not yet been fully elucidated since most findings were from type 1 DM, whereas type 2 DM is far more complicated than type 1 DM. Except for hyperglycemia, a series of other metabolic abnormalities such as insulin resistance (IR), hypertension, hyperlipidemia, and obesity are common in type 2 DM. Therefore, the mechanism of renal injury is complicated in patients with type 2 DM.

Among the main pathophysiological characteristics of type 2 DM, IR and compensatory hyperinsulinemia occur long before clinical diabetes. Hyperinsulinemia impairs insulin activity and metabolic signaling pathways and also affects the structure and function of the tissues and organs including the kidney. IR/hyperinsulinemia is an independent risk factor for chronic kidney diseases [8, 9]. Hyperinsulinemia leads to hypertension by aggravating the water-sodium retention of renal tubules. The resulted relaxation and contraction dysfunction of endothelial cells causes tubulointerstitial ischemia and hypoxia. Hyperinsulinemia also participates in renal tubular cell proliferation that induces tubulointerstitial remodeling.

However, whether and how hyperinsulinemia play roles in the early damages of kidney before DM remain elusive. We dynamically studied the changes of the structure and function of renal tissue in different stages of type 2 DM (NGT, IGT, DM, and DKD stages) using OLETF rats. We also studied the effects of different concentrations of the insulin and IRS-1/PI3-K/Akt pathway on albumin reabsorption in a rat proximal renal tubular epithelial cell line (NRK-52E).

## 2. Materials and Methods

### 2.1. Animal Experiments and Methods

This study was performed in strict accordance with the NIH guidelines for the care and use of laboratory animals (NIH Publication No. 85-23 Rev. 1996) and was approved by the Institutional Animal Care and Use Committee of the Tianjin Medical University.

#### 2.1.1. Animals and Grouping

32 male OLETF rats, a model of spontaneous type 2 DKD, were supplied by Otsuka Pharmaceutical Co., Ltd. (Tokushima, Japan). OLETF rats were fed a high-fat diet and were housed in an air-conditioned room at 20°C-25°C with 50%-70% humidity and a 12 h light-dark cycle. Rats were allowed free access to food and water. 32 male LETO rats were used as the nondiabetic model contrasted to OLETF rats.

**Figure 1:**
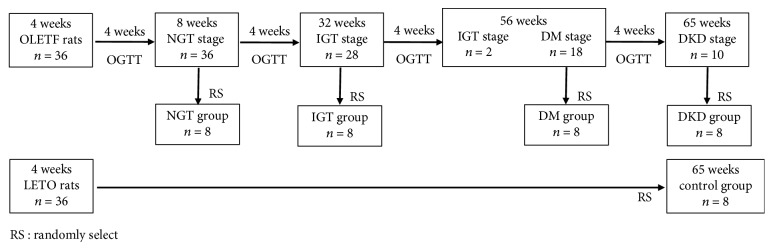
The grouping steps in animal experiment.

An oral glucose tolerance test (OGTT) was performed every 4 weeks. Before the test, the rats were fasted for 15 hours overnight and were administered to 30% glucose solution (2 g/kg) by gastric gavage. Blood samples were taken from the tail vein before glucose loading and 30, 60, and 120 min after glucose loading. Blood glucose levels were determined by an automatic blood glucose analyzer. Plasma insulin concentrations were detected by radioimmunoassay. OLETF rats were classified into three stages on the basis of the OGTT results [10]: the NGT stage, which had a normal glucose level; the IGT stage, which had a peak level of blood glucose > 16.8 mmol/L or 120 min blood glucose level > 11.1 mmol/L; the DM stage, when both of the above two conditions were observed in rats. The DKD stage was defined as the amount of 24-hour urinary microalbumin (24 h UMA) in OLETF rats which was significantly greater than that in LETO rats. Eight OLETF rats were randomly selected and euthanized, respectively, in their NGT, IGT, DM, and DKD stages as the NGT, IGT, DM, and DKD groups. Eight LETO rats, which had the same age with the DKD group, were treated as the control group. The grouping steps are shown in Figure 1.

#### 2.1.2. Renal Function and Other Biochemical Markers

Rats were placed in metabolic cages. 24 h urine and random urine were collected. 24 h UMA, retinol-binding protein (RBP), N-acetyl-*β*-glucosaminidase (NAG), and *β*2-microglobulin (*β*2-MG) were measured by a Roche analyzer. Cystatin C (CysC) and neutrophil gelatinase-associated lipocalin (NGAL) were measured by enzyme-linked immunosorbent assay (ELISA).

Arterial blood was collected from the femoral artery. Blood urea nitrogen (BUN), serum creatinine (Scr), total cholesterol (TC), triglyceride (TG), and free fatty acid (FFA) were tested by an automatic biochemical analyzer. Tumor necrosis factor-*α* (TNF-*α*) and interleukin-6 (IL-6) were tested by ELISA.

#### 2.1.3. Renal Structure

*(1) Immunohistochemistry (IHC)*. Renal tissues were immediately fixed in 4% formalin and subsequently embedded in paraffin. The expression of IRS-1 (ab131487, abcam), pSer IRS-1 (AF3272, Affinity), megalin (Santa Cruz, Sc-25470), and cubilin (Santa Cruz, Sc-23644) in renal tubules and interstitium were detected by IHC.

*(2) Light Microscope*. Renal tissues were fixed in 4% paraformaldehyde and embedded in paraffin. Tissue slices were cut at 4 *μ*m thickness, dewaxed in xylene, rehydrated in decreasing concentrations of ethanol in water and stained by H&E, and then examined by a light microscope.

*(3) Transmission Electron Microscope*. Renal tissues were immediately placed in fixative (2.5% glutaraldehyde and 1% osmium tetraoxide), dehydrated using graded alcohol and epoxypropane, and then embedded in Epon 812. Ultrathin sections (50 *μ*m±) were cut using an ELICA ULTRACUT-R ultramicrotome and stained with uranyl acetate and lead citrate. The sections were examined using a HITACHI-7500 transmission electron microscope.

### 2.2. Cell Experiments

#### 2.2.1. Cells and Medium

Proximal tubular epithelial cells from a Rattus norvegicus kidney (NRK-52E) were maintained in DMEM (1 g/L glucose, Hyclone) supplemented with 10% fetal bovine serum (Hyclone)+penicillin/streptomycin (Gibco) at 37°C and 5% CO_2_. Cells at 3-5 passages were used for the experiments.

#### 2.2.2. Cell Experiment Methods

*(1) Cell Viability*. MTT is 3-(4,5-dimethylthiazole-2)-2,5-dimethyl tetrazolium bromine blue. Succinodehydrogenase in living cells can make MTT decompose to formazan, a purple dye. The amount of formazan is proportional to the number and activity of living cells. The number of formazan can be dissolved by solvent, and the absorbance value (OD value) can be measured by an enzyme-labeling instrument to indirectly reflect the number and activity of cells. The effect of the best insulin concentration on the activity of NRK-52E cells was screened by this method.

Cell suspensions were seeded in a 96-well plate with 4000 cells in each well. NRK-52E cells were treated with indicated concentrations of insulin (0, 1, 5, 10, 50, 100, 103, 5 × 10^3^, 10^4^ ng/mL). After 24 h, cells were treated with MTT for 4 h and then were shaken for 10 min to fully dissolve the crystals after being treated by 150 *μ*L DMSO in each well. The OD values for cells were read in 570 nm with a microplate reader. Experiments were repeated for 3 times.

*(2) Expression of Megalin and Cubilin*. Immunofluorescence method and fluorescence quantitative RT-PCR were used to assess the expression of megalin (Rabbit polyclonal to Ras, Santa Cruz) and cubilin (Goat polyclonal to Ras, Santa Cruz) in NRK-52E cells after being stimulated by different concentrations of insulin for 24 hours.

*(3) Reabsorption of Albumin*. A laser scanning confocal microscope was used to assess the intake of tetramethylrhodamine isothiocyanate-labeled bovine serum albumin (TRITC-BSA, sigma) in NRK-52E cells.

Cell suspensions were seeded in a 24-cell plate with 3000 cells in each cell. After being treated by different concentrations of insulin, NRK-52E cells were treated with TRITC-BSA (500 *μ*g/mL) for 4 hours. The albumin absorption by NRK-52E cells was evaluated through the assessment of the red fluorescence intensity of TRITC-albumin by a laser confocal fluorescence microscope (excitation wavelength and emission wavelength were 540 nm and 570 nm, respectively).

*(4) Role of IRS-1/PI3-K/Akt Signaling Pathway in the Reabsorption of Albumin in Renal Tubular Epithelial Cells*. Fluorescence quantitative RT-PCR and western blot were used to detect the expression of IRS-1 (rabbit polyclonal to Ras, abcam), PI3-K (PI3-K p85, rabbit, Proteintech), and Akt (rabbit polyclonal to Ras, abcam) after cells were treated with different concentrations of insulin.

We further treated cells with 5 ng/mL insulin (CN group), insulin combined with a PI3-K inhibitor (5 ng/mL insulin+LY294002, CN+LY group), and 50 ng/mL insulin (high-insulin group) to check the intake of TRITC-albumin as well as the expression of megalin and cubilin in NRK-52E cells.

### 2.3. Statistical Analysis

Statistical analysis was performed using the IBM SPSS Statistics 20.0 software. All normally distributed data were expressed as the mean ± SD, and all other data were expressed as the median (range). Differences among the three groups were analyzed by one-way analysis of variance for normally distributed continuous parameters. If differences were found, the LSD *t*-test was used for the comparison between two groups. Nonnormal distributed data were compared by the Kruskal-Wallis test. If differences were found, the Mann-Whitney test was used for the comparison between two groups. A chi-square test was used in comparing the differences of measurement data. Results were considered statistically significant if the two-tailed *P* value was < 0.05.

## 3. Results

### 3.1. Change of Biochemical Markers in Different Stages of Type 2 DM

#### 3.1.1. Fasting Blood Glucose (FBG)

Compared with that in the control group, the FBG level in the NGT and IGT groups did not change. In the DM and DKD groups, the FBG level was increased and was higher than that of the other three groups (*P* < 0.05) (Table 1).

#### 3.1.2. Fasting Insulin (FINS)

Compared with that in the control group, the FINS level in the NGT group did not change. However, the FINS level was increased at the IGT group and was higher than that of the control group (*P* < 0.05). Although the FINS levels began to fall in the DM and DKD groups, they were still higher than that in the control group (*P* < 0.05) (Table 1).

#### 3.1.3. Blood Lipid and Weight

Compared with the control group, the blood lipids including TG, TC and FFA did not change in the NGT group, but were increased in the IGT and DM groups (P < 0.05) (Table 1).

Compared with that in the control group, the weight in the NGT group did not change. In the IGT group, the weight was higher than that in the control and NGT groups (*P* < 0.05). However, in the DM and DKD groups, the weight decreased gradually which was not different from that of the IGT group but was still higher than those of the control and NGT groups (*P* < 0.05) (Table 1).

### 3.2. Pathophysiological Characteristics of Nephropathy in the IGT Stage in OLETF Rats

#### 3.2.1. Tubular Function

The levels of NAG and NGAL which represent the injury of tubular epithelium and the levels of *β*2-MG, RBP, and CysC which represent tubular reabsorption function did not change between the NGT group and the control group. However, all markers were increased in the IGT, DM, and DKD groups except *β*2-MG, which was increased later at the DM and DKD groups (*P* < 0.05) (Table 2).

#### 3.2.2. Glomerular Function

24 h UMA, Scr, and BUN between the NGT and IGT groups were not different. However, in the DM group, the Scr but not 24 h UMA or BUN was higher than those in the NGT and control groups. In the DKD group, 24 h UMA was higher than those in the other four groups (Table 3).

#### 3.2.3. Pathological Changes of Renal Tubule

**Figure 2:**
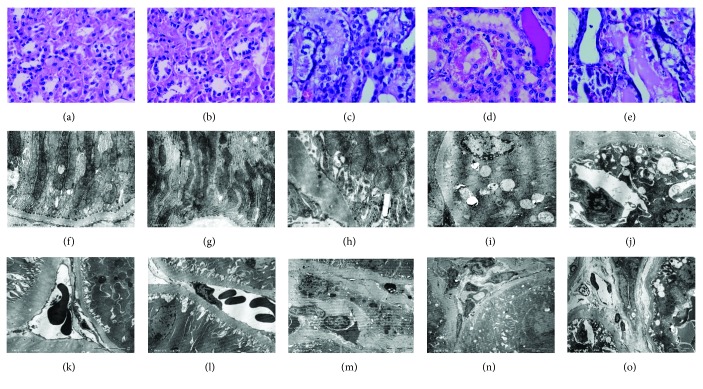
Pathological changes of the renal tubule. Light microscope: (a) the control group by H&E (×400), (b) the NGT group by H&E (×400), (c) the IGT group by H&E (×400), (d) the DM group by H&E (×400), and (e) the DKD group by H&E (×400). Transmission electron microscope: (f) Basal side of the renal tubule in the control group (×30000), (g) basal side of the renal tubule in the NGT group (×30000), (h) basal side of the renal tubule in the IGT group (×30000), (i) basal side of the renal tubule in the DM group (×10000), (j) basal side of the renal tubule in the DKD group (×10000), (k) the renal interstitium in the control group (×10000), (l) renal interstitium in the NGT group (×7000), (m) the renal interstitium in the IGT group (×60000), (n) the renal interstitium in the DM group (×3000), and (o) the renal interstitium in the DKD group (3500).

Compared with those in the control group, the rats in the NGT group had normal tubular morphology as well as ultrastructures (Figures 2(a), 2(b), 2(f), 2(g), 2(k), and 2(l)). However, in the IGT group, significant damages had occurred to renal tubules (Figure 2(c)). The brush border of tubular epithelial cells in the cortex was shed off. There were some vacuole granules and necrosis in the cortex and medulla. Inflammatory cells had infiltrated in the tubulointerstitium. Mild thickening in the arterial wall had been noticed. Under electron microscopy, obvious changes had also been found, including the irregular arrangement of renal tubular cells, thickening of the tubular basement membrane, disordered tubular folds, degradation in part of the mitochondrial inner and outer membranes, the homogeneous and flocculent alteration in part of the mitochondrial ridge and matrix after degradation, and the deposition of glycogen granules near mitochondria (Figure 2(h)). Moreover, an expansion of the space between the inner and outer nuclear membranes of tubulointerstitial vascular endothelial cells, stasis of intravascular red blood cells, and infiltration of interstitial inflammatory cells were also found (Figure 2(m)). In the DM group, under a light microscope (Figure 2(d)), renal tubular atrophy with apparent vacuole granules and necrosis were found in both the cortex and medulla. Protein casts began to appear in the tubule. Serious infiltrations of inflammation and interstitial fibrosis as well as the thickening of the arterial wall were noticed. Ultrastructural observation revealed a uniform thickening of the renal tubular basement membrane, slight tortuosity of the renal tubular cell nucleus, an increase of vacuoles in cells, swelling of a part of the mitochondria, degradation of the most inner and outer membranes and the ridge, remaining of the residual ridge and membrane degradation residues (Figure 2(i)), and rupture of the tubulointerstitial vascular basement membrane (Figure 2(n)). In the DKD group, under a light microscope (Figure 2(e)), thickening of the renal tubular basement membrane, more aggravated necrosis in the cortex and medulla, numerous protein casts, focal hyperplasia of interstitial fibrous tissues, infiltration of inflammatory cell, and thickening of the arterial wall were found. Ultrastructural observation exhibited an enlarged space between renal tubular cells, irregular arrangement of cells, many vacuoles and lysosomes, shallow tubular folds, thickening, stratification and wrinkling of the basement membrane with granular sediments, fuzziness and irregularity of tubular mitochondria, accumulation of glycogen particles (Figure 2(j)), tubulointerstitial edema, infiltration of inflammatory cell, and discontinuous damage of endothelial cell (Figure 2(o)).

#### 3.2.4. Pathological Changes of Glomerulus

**Figure 3:**
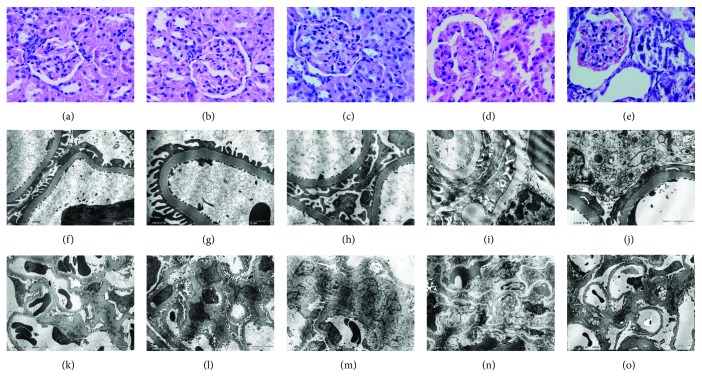
Pathological changes of glomerulus. Light microscope: (a) the control group by H&E (×400), (b) the NGT group by H&E (×400), (c) the IGT group by H&E (×400), (d) the DM group by H&E (×400), and (e) the DKD group by H&E (×400). Transmission electron microscope: (f) the glomerular filtration membrane in the control group (×20000), (g) the glomerular filtration membrane in the NGT group (×25000), (h) the glomerular filtration membrane in the IGT group (×20000), (i) the glomerular filtration membrane in the DM group (×20000), (j) the glomerular filtration membrane in the DKD group (×20000), (k) the mesangial area in the control group (×5000), (l) the mesangial area in the NGT group (×5000), (m) the mesangial area in the IGT group (×5000), (n) the mesangial area in the DM group (×5000), and (o) the mesangial area in the DKD group (×3500).

Compared with those in the control group, rats in the NGT group had normal glomerular morphology as well as ultrastructures (Figures 3(a), 3(b), 3(f), 3(g), 3(k), and 3(l)). In the IGT group, there were slight structure change (Figure 3(c)), i.e., a minor expansion of the volume and mild hyperplasia of mesangial cells. Ultrastructural analysis found that the glomerular filtration membrane had a uniform thickness and a clear three-layered structure. Endothelial cells and podocytes had good structures with uniformly distributed foot processes (Figure 3(h)). The mesangial area was enlarged. The cell number did not increase while the cell volume as well as the secretion of mesangial matrix was increased slightly (Figure 3(m)). In the DM group (Figure 3(d)), the glomerular basement membrane was thickened, the mesangial area was widened, and the mesangial cell number was increased. Ultrastructural analysis found that there were thickening of the glomerular filtration membrane, spherical protruding in some parts of the basement membrane, and fusion of the foot process (Figure 3(i)). The mesangial area was expanded, the number of mesangial cells was increased, and the mesangial matrix was extended in a cord shape (Figure 3(n)). In the DKD group (Figure 3(e)), there were thickening of the glomerular basement membrane, extending of the mesangial area, proliferation of the mesangial cell, and occasional segmental sclerosis. An ultrastructural study disclosed a thickening of the glomerular filtration membrane, fusion and loss of the most foot processes, swelling and degradation of podocyte, an expansion of the rough endoplasmic reticulum cisterna, a swelling of mitochondria, and a degranulation phenomenon (Figure 3(j)). The mesangial area was expanded, the number of mesangial cells was increased, and the secretion of the mesangial matrix was unregulated (Figure 3(o)). Cells containing a large number of fat vacuoles were found in the mesangial area, and some cells contained phagocytic substances and terminal lysosomes (Figure 3(o)).

### 3.3. IRS-1 and pSer IRS-1 Expression in the IGT Stage of OLETF Rats

**Figure 4:**
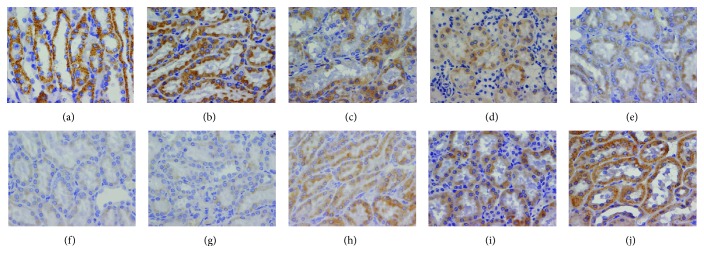
Expression of IRS-1 and pSer IRS-1 by immunohistochemistry in a light microscope: (a) IRS-1 in the control group (×400), (b) IRS-1 in the NGT group (×400), (c) IRS-1 in the IGT group (×400), (d) IRS-1 in the DM group (×400), (e) IRS-1 in the DKD group (×400), (f) pSer IRS-1 in the control group (×400), (g) pSer IRS-1 in the NGT group (×400), (h) pSer IRS-1 in the IGT group (×400), (i) pSer IRS-1 in the DM group (×400), and (j) pSer IRS-1 in the DKD group (×400).

IRS-1 was highly expressed in renal tubular epithelial cells in the NGT group, while pSer IRS-1 was expressed at a low level. Both IRS-1 and pSer IRS-1 were not different from that in the control group. In the IGT group, IRS-1 began to decrease and was lower than that in the control group and the NGT group (*P* < 0.05). pSer IRS-1 began to increase and was higher than that in the control group and the NGT group (*P* < 0.05). The expression of IRS-1 continued to decrease and was further lower in the DKD group than that in the DM group (*P* < 0.05), while the expression of pSer IRS-1 continued to increase and was higher in the DKD group than that in the DM group (*P* < 0.05) (Table 4 and Figure 4).

### 3.4. Megalin and Cubilin Expression in the IGT Stage of OLETF Rats

**Figure 5:**
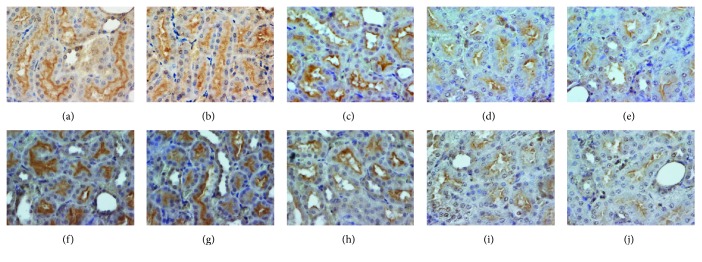
Expression of megalin and cubilin by immunohistochemistry in a light microscope: (a) megalin in the control group (×400), (b) megalin in the NGT group (×400), (c) megalin in the IGT group (×400), (d) megalin in the DM group (×400), (e) megalin in the DKD group (×400), (f) cubilin in the control group (×400), (g) cubilin in the NGT group (×400), (h) cubilin in the IGT group (×400), (i) cubilin in the DM group (×400), and (j) cubilin in the DKD group (×400).

In the NGT group, megalin and cubilin were highly expressed on the luminal side of the renal tubular epithelial cells and were not different from those of the control group. In the IGT group, both megalin and cubilin were decreased compared to those in the control group and the NGT group (*P* < 0.05). Further reduced expressions of megalin and cubilin were noticed in the DM and DKD groups, but there was no difference between these two groups (*P* > 0.05) (Table 4 and Figure 5).

### 3.5. Effect of Different Concentrations of Insulin on the Expression of Megalin and Cubilin and TRITC-Albumin Reabsorption in NRK-52E Cells

**Figure 6:**
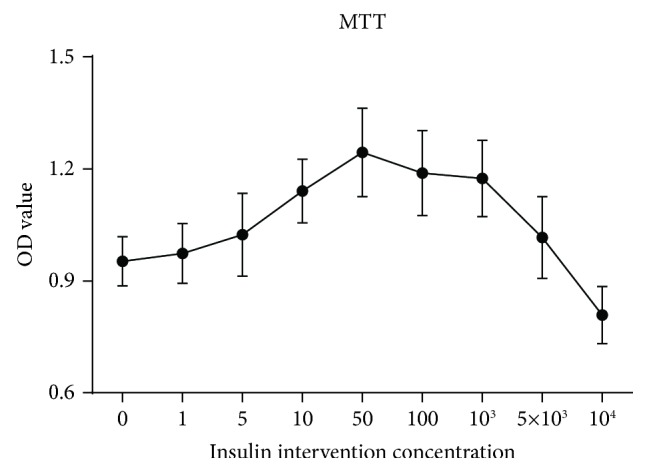
Effect of different concentrations of insulin on cell viability by MTT.

Combined with physiological concentration range 0-5 ng/mL and MTT results (Figure 6), we used different concentrations of insulin in this study: 0 ng/mL (the control group), 5 ng/mL (the low-concentration group, equivalent of physiological concentration of insulin), 10 ng/mL (the moderate concentration group), and 50 ng/mL (the high-concentration group).

**Figure 7:**
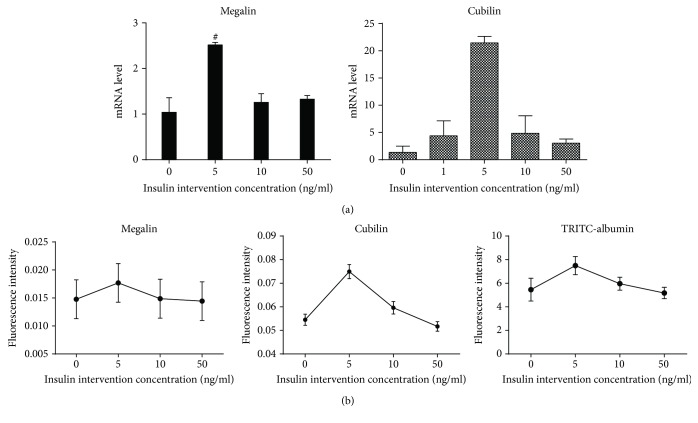
Different concentrations of insulin on the expression of megalin and cubilin in NRK-52E cells and their effects on TRITC-albumin reabsorption. (a) The mRNA level of megalin and cubilin of NRK-52E cells in indicated concentrations of insulin. (b) The expression of megalin and cubilin and the reabsorption of TRITC-BSA in indicated concentrations of insulin.

NRK-52E cells were treated with indicated concentrations of insulin (0, 5, 10, and 50 ng/mL). After 24 h, we found that the mRNA levels of megalin and cubilin were the highest in cells treated with 5 ng/mL insulin, which was different from that in the control group (*P* < 0.05). However, along with the increase of insulin concentration, the expressions of megalin and cubilin were decreased and the lowest expression was found in the group with the highest insulin concentration (Figure 7).

**Figure 8:**
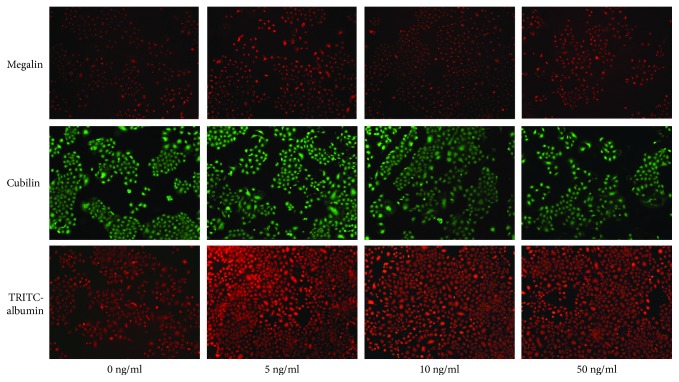
Effects of different concentrations of insulin on the expression of megalin and cubilin and TRITC-albumin reabsorption in NRK-52E cells.

The reabsorption of TRITC-BSA by NRK-52E cells was the highest in cells treated with 5 ng/mL insulin, which was different from that in the control group. However, along with the increase of insulin intervention concentration, the uptake of TRITC-BSA by cells decreased and the least was in the highest insulin concentration group. Therefore, the expressions of megalin and cubilin in renal tubular epithelial cells were inconsistent with its albumin reabsorption function when treated by different levels of insulin. High concentration of insulin inhibited the expression of megalin and cubilin and TRITC-albumin reabsorption in NRK-52E cells (Figure 8).

### 3.6. The Expression of IRS-1, PI3-K, and Akt in NRK-52E Cells Treated by Different Concentrations of Insulin

**Figure 9:**
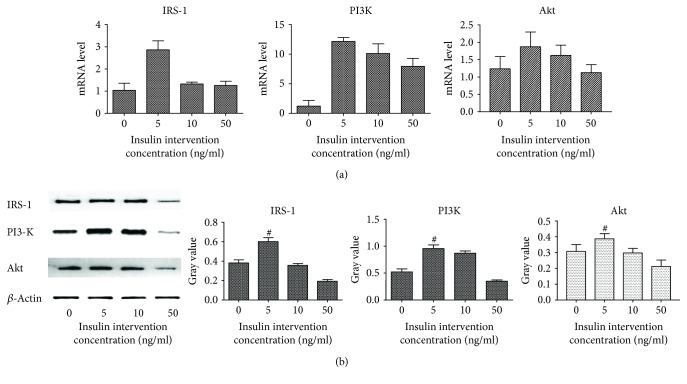
Changes of the mRNA and the protein level of IRS-1/PI3-K/Akt after being treated by different concentrations of insulin. (a) The mRNA level of IRS-1, PI3-K, and Akt of NRK-52E cells in indicated concentrations of insulin. (b) The expression of IRS-1, PI3-K, and Akt in indicated concentrations of insulin.

The mRNA levels of IRS-1, PI3-K, and Akt were the highest in cells treated with 5 ng/mL insulin and were significantly different from those in the control group (*P* < 0.05). However, along with the increase of insulin concentration, the expressions of IRS-1, PI3-K, and Akt were gradually decreased. The protein levels of IRS-1, PI3-K, and Akt detected by western blot were in accordance with the mRNA levels. These results suggested that the INS/IRS-1/PI3-K/Akt signaling pathway was restrained by high concentration insulin in NRK-52E cell (Figure 9).

### 3.7. Comparison between the PI3-K Inhibitor and the High Concentration of Insulin on the Expression of Megalin and Cubilin and TRITC-Albumin Reabsorption in NRK-52E Cells

**Figure 10:**
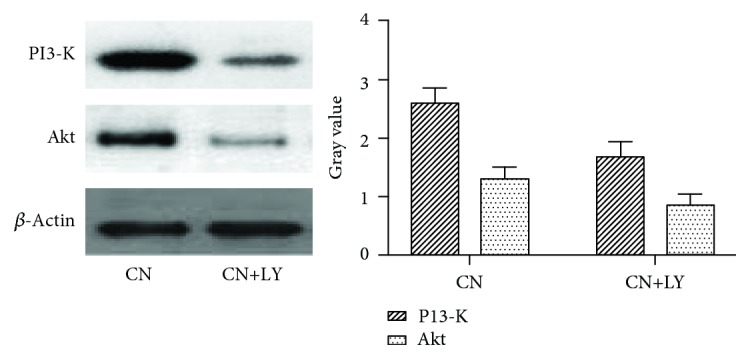
The inhibitory effect of the PI3-K inhibitor on the PI3-K/Akt signaling pathway.

After the NRK-52E cells were treated with the PI3-K inhibitor (LY294002), we further detected the expression of Akt to illustrate the effect of the PI3-K inhibitor on PI3-K/Akt insulin signaling pathway. The results showed that the expression of Akt in the CN+LY group was significantly lower than that in the control group. Therefore, the PI3-K inhibitor inhibited the PI3-K/Akt signaling pathway (Figure 10).

**Figure 11:**
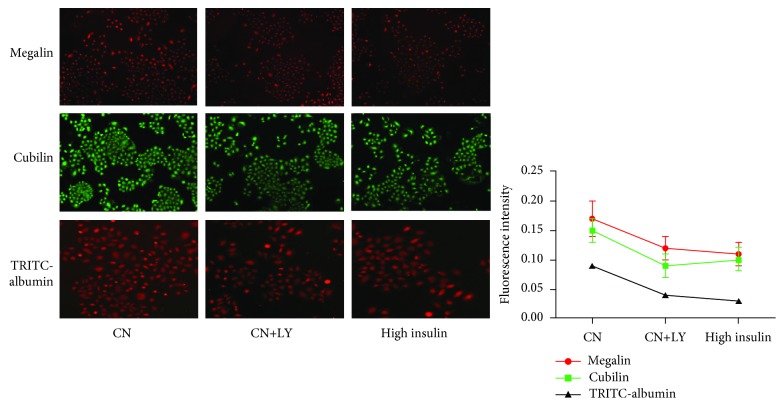
Comparison between the PI3-K inhibitor and the high concentration of insulin on the expression of megalin and cubilin and TRITC-albumin reabsorption in NRK-52E cells.

The expression of megalin and cubilin and TRITC-albumin reabsorption in the CN+LY group and in the high insulin group were significantly lower than those of the control group (*P* < 0.05). The results suggested that both the inhibitor of the insulin signaling pathway and the high concentration of insulin could inhibit the expressions of megalin and cubilin as well as the reabsorption of TRITC-albumin in NRK-52E cells (Figure 11).

## 4. Discussion

OLETF rat, which manifests by obese, insulin resistance, spontaneous hyperglycemia, and proteinuria after 30 weeks, is an ideal animal model of DKD in human [11]. In this study, OLETF rats were given a high-fat diet, and their blood glucose, blood lipid, and body weight were gradually increased. There was transient hyperinsulinemia in the IGT stage, and the insulin levels were gradually dropped after IGT. After week 56, the blood glucose after the OGTT test meets the diagnostic criteria for diabetes. Therefore, type 2 DM was established successfully in OLETF rats. Proteinuria occurred in OLETF rats after 65 weeks. Simultaneously, there were pathological changes such as thickening of the glomerular basement membrane, expanding of the mesangial area, proliferation of the mesangial cell, and sclerosis in focal segmental glomeruli. All were in accordance with type 2 DKD in human.

We also found significant renal structure damage even in the IGT group, especially renal tubules and interstitial blood vessels. Under a light microscope, the cortical brush borders of tubular epithelial cells were shed off, vacuole granules and necrosis were found in cells, inflammatory cells infiltrated in the interstitium, and mild thickening of the arterial wall was also found. Ultrastructural observation also revealed significant damage of tubular epithelial cells as well as endothelial cells. In accordance to the structure alteration, we also found a significant impairment of renal tubular function even in the IGT group of OLETF rats. NAG (or NGAL) which reflects the injury of the renal tubular epithelial cells and RBP (or CysC) which reflects the injury of renal tubular reabsorption were increased in the IGT group. In DM group, the above-mentioned renal tubular injuries were aggravated and accompanied by a series of pathophysiological changes, such as thickening of the glomerular basement membrane, widening of the mesangial area, proliferation of mesangial cell, and increasing of 24 h UMA. In contrast to the traditional opinion about DKD, kidney disease in the IGT stage manifests by renal tubular damage. Therefore, we propose this renal tubular damage which occurs in the IGT stage of diabetes as “IGT nephropathy.” Accumulating evidences have demonstrated that in the early stage of DKD, glomeruli are not changed while the structure and function of the renal tubule have been damaged [12]. In a clinical practice, it is found that patients with either type 1 or type 2 diabetes have significantly higher levels of urine NAG, *α*1-MG, *β*2-MG, and RBP compared to the NGT subjects [13, 14]. This occurs even when they have normal MAU. Our previous clinical studies have also confirmed that 78.2% diabetes patients have various degrees of renal tubular dysfunction even without proteinuria [4]. There are other studies which found that MAU also occurs in the IGT patients. This can be attributed to the decreased reabsorption of urine albumin owing to the dysfunction of renal tubular epithelial cells but not the damage of the glomerular filtration barrier [15].

What is the pathogenesis of nephropathy in the IGT stage? Different from type 1 DM, type 2 DM has prolonged pathophysiological progress. As a crucial prediabetes stage, IGT not only is closely related to the onset of cardiac, cerebral, and other macrovascular diseases but also is an independent risk factor for microvascular complications such as DKD [16]. IR and compensatory hyperinsulinemia are two key pathophysiological changes of IGT. In our study, the fasting insulin level of OLETF rats increased significantly in IGT group. In the diabetes and DKD groups, although the insulin levels decreased compared to that in the IGT group, they were still higher than that in the NGT group. This compensatory hyperinsulinemia is harmful to tissues such as kidney. In one study, it is found that the damages of high insulin to the kidney begins before the rising of blood glucose [8]. Other studies have showed that hyperinsulinemia is closely associated not only with endothelial cell dysfunction, hypertension, and kidney disease [17–20] but also with the onset of low-level albuminuria [21–23]. It is traditionally believed that MAU is a marker of early DKD which reflects the damage of glomeruli [24]. However, recently, the onset of MAU during IGT stage has been associated with the reduced reabsorption of albumin by renal tubules rather than high glomerular filtration [15].

Megalin and cubilin are multifunctional receptor glycoproteins which play important roles in mediating renal protein reabsorption. Cubilin-deficient animal models are subjected to significant proteinuria. Moreover, proteinuria occurring in patients with Fanconi syndrome can be explained by the lack of megalin and cubilin. The expressions of megalin and cubilin are affected by a variety of factors including their ligand insulin, but whether they are different under circumstances with different insulin levels remains unclear. In our study, we found that the expressions of megalin and cubilin were increased mostly by the low level of insulin. Laser confocal microscopy also displayed that NRK-52E cells treated by low-concentration insulin had the highest uptake of albumin. In the high-concentration intervention group, however, both the expressions of megalin and cubilin and the uptake of albumin were significantly decreased. Therefore, insulin at physiological concentration promotes megalin and cubilin expression and facilitates the uptake of albumin by renal tubular epithelial cells. However, with the increase of insulin concentration, the expressions of the two receptors were gradually decreased, and the reabsorption of albumin by renal tubular epithelial cells was also reduced. Therefore, insulin at high concentrations exert significant inhibitory effects.

As multifunctional receptor glycoproteins, megalin and cubilin can bind to various ligands and then mediate the absorption function. Megalin and cubilin play key roles in the renal reabsorption of albumin, RBP, CysC, and so on. The dysfunction of megalin and cubilin will cause reabsorption disorders of small-molecule proteins leading to the appearance of these proteins in urine. In our study, the urine RBP and CysC of OLETF rats in the IGT group were increased. Since the insulin level of IGT rats were increased, will it affect the reabsorption function of renal tubular epithelial cells?

We treated renal tubular epithelial cells with different concentrations of insulin to investigate its effect on megalin/cubilin expression and on albumin reabsorption. We found that in the presence of insulin at physiological concentration, the insulin signaling pathway IRS-1/PI3-K/Akt in renal tubular epithelial cells was highly activated, and the expressions of megalin and cubilin were highly upregulated which was accompanied by a maximum uptake of albumin. We found that both the inhibitor of the insulin signaling pathway and the high concentration of insulin could inhibit the expressions of megalin and cubilin as well as the reabsorption of TRITC-albumin in NRK-52E cells. Therefore, we speculated that high concentration of insulin affected the expressions of megalin and cubilin possibly via the IRS-1/PI3-K/Akt signaling pathway, which caused the disturbance of albumin endocytosis and finally led to proteinuria.

In summary, kidney damage occurs early in the IGT stage of diabetic rats. In contrast to DKD, we named this kind of renal structural damage and functional changes as “IGT kidney disease.” By inhibiting the IRS-1/PI3-K/Akt signaling pathway, high concentration of insulin downregulates the expression of megalin and cubilin, which then causes the reabsorption dysfunction of albumin by renal tubular epithelial cells leading to albuminuria. However, whether hyperinsulinemia plays a leading role in vivo remains to be proved by further studies and more inhibitors should be used.

## Figures and Tables

**Table 1:** Alteration of biochemical markers in different stages of type 2 DM.

Group	FBG (mmol/L)	FINS (uIU/L)	FFA (mmol/L)	TG (mmol/L)	TC (mmol/L)	Weight (g)
Control	5.74 ± 0.21	23.00 ± 1.60	0.85 ± 0.16	0.61 ± 0.18	1.71 ± 0.17	293.74 ± 15.95
NGT	5.60 ± 0.29	23.37 ± 3.08	0.85 ± 0.20	0.64 ± 0.16	1.78 ± 0.16	309.67 ± 14.03
IGT	5.90 ± 0.33	62.62 ± 4.32^ab^	1.13 ± 0.35^ab^	1.09 ± 0.28^ab^	2.08 ± 0.39^ab^	593.46 ± 49.92^ab^
DM	6.71 ± 1.51^abc^	33.53 ± 4.71^abc^	1.22 ± 0.40^ab^	1.35 ± 0.46^ab^	2.36 ± 0.38^ab^	579.33 ± 51.99^ab^
DKD	6.83 ± 0.41^abc^	29.35 ± 3.13^abcd^	1.30 ± 0.40^ab^	1.40 ± 0.44^abc^	3.02 ± 0.45^abcd^	563.14 ± 88.92^ab^

*P*	0.003	0.001	0.014	0.001	0.001	0.001

^a^ *P* < 0.05 vs. the control group. ^b^*P* < 0.05 vs. NGT. ^c^*P* < 0.05 vs. IGT. ^d^*P* < 0.05 vs. DM.

**Table 2:** Alteration of tubular function marker in different stages of type 2 DM.

Group	NAG (U/L)	NGAL (ng/mL)	*β*2-MG (mg/L)	RBP (mg/L)	CysC (ng/mL)
Control	42.50 ± 4.07	44.28 ± 6.63	0.014 ± 0.006	0.100 ± 0.053	32.12 ± 4.09
NGT	42.29 ± 5.08	46.46 ± 6.97	0.013 ± 0.005	0.093 ± 0.058	31.93 ± 2.70
IGT	52.70 ± 5.61^ab^	59.81 ± 12.51^ab^	0.018 ± 0.007	0.305 ± 0.030^ab^	38.00 ± 1.82^ab^
DM	62.97 ± 9.21^abc^	72.66 ± 13.78^abc^	0.030 ± 0.009^abc^	0.581 ± 0.144^abc^	41.83 ± 2.09^abc^
DKD	70.04 ± 9.65^abc^	80.19 ± 15.03^abc^	0.036 ± 0.011^abc^	0.980 ± 0.195^abcd^	45.65 ± 2.66^abcd^

*P*	0.001	0.001	0.001	0.001	0.001

^a^ *P* < 0.05 vs. the control group. ^b^*P* < 0.05 vs. NGT. ^c^*P* < 0.05 vs. IGT. ^d^*P* < 0.05 vs. DM.

**Table 3:** Alteration of glomerular function marker in different stages of type 2 DM.

Group	24 h UMA (*μ*g/24 h)	Scr (*μ*mol/L)	BUN (mmol/L)
Control	151.68 ± 24.22	32.53 ± 2.51	6.75 ± 0.55
NGT	148.45 ± 22.95	31.80 ± 1.16	6.88 ± 1.05
IGT	167.98 ± 24.37	33.75 ± 2.76	7.16 ± 1.26
DM	180.55 ± 29.43	36.02 ± 3.25^ab^	7.55 ± 1.21
DKD	224.22 ± 57.88^abcd^	38.18 ± 5.46^abc^	8.45 ± 1.25^abc^

*P*	0.001	0.001	0.008

^a^ *P* < 0.05 vs. the control group. ^b^*P* < 0.05 vs. NGT. ^c^*P* < 0.05 vs. IGT. ^d^*P* < 0.05 vs. DM.

**Table 4:** Semiquantitative analysis of IRS-1, pSer IRS-1, megalin, and cubilin in renal tubule and interstitium.

Group	IRS-1	pSer IRS-1	Megalin	Cubilin
Control	0.22 ± 0.04	0.09 ± 0.03	0.26 ± 0.04	0.24 ± 0.04
NGT	0.22 ± 0.04	0.09 ± 0.02	0.25 ± 0.04	0.24 ± 0.03
IGT	0.18 ± 0.03^ab^	0.13 ± 0.03^ab^	0.18 ± 0.03^ab^	0.19 ± 0.04^ab^
DM	0.13 ± 0.03^abc^	0.17 ± 0.03^abc^	0.14 ± 0.03^abc^	0.15 ± 0.03^abc^
DKD	0.09 ± 0.02^abcd^	0.21 ± 0.04^abcd^	0.12 ± 0.04^abc^	0.14 ± 0.03^abc^

*P*	0.001	0.001	0.001	0.001

^a^ *P* < 0.05 vs. the control group. ^b^*P* < 0.05 vs. NGT. ^c^*P* < 0.05 vs. IGT. ^d^*P* < 0.05 vs. DM.

## Data Availability

The data used to support the findings of this study are available from the corresponding author upon request.

## Supplementary Materials

Supplementary Materials Primer sequences of target genes as supplements are shown in the table.
