# Supplementary material for: Hyperinsulinemia Can Cause Kidney Disease in the IGT Stage of OLETF Rats via the INS/IRS-1/PI3-K/Akt Signaling Pathway
Source: J Diabetes Res. 2019 Oct 13;2019:4709715. doi: 10.1155/2019/4709715 (PMC6815570; doi:10.1155/2019/4709715)
Supplement: Supplementary Materials — Primer sequences of target genes as supplements are shown in the table. [file 4709715.f1.docx]

Primer sequences of target genes as supplements were shown in the table.

Table Primer sequence and Amplification length of the Target genes

| Target fragment | Primer sequence | | Fragment length |
| --- | --- | --- | --- |
| Rat-Megalin | F | 5’-CCGCAAAATGATAGCCCAGC-3’ | 203 bp |
|  | R | 5’-TGATGGCATTGGGCCATTCT-3’ |  |
| Rat-Cubilin | F | 5’-ACAGCACAGACTGTACACGG-3’ | 176bp |
|  | R | 5’-GGCACGGAAACCCTCAAAAC-3’ |  |
| Rat-GAPDH | F | 5’-GCAAGTTCAACGGCACAG-3’ | 140 bp |
|  | R | 5’-GCCAGTAGACTCCACGACAT-3’ |  |

| Target fragment | Primer sequence | | Fragment length |
| --- | --- | --- | --- |
| Rat-GAPDH | F | 5’-GCAAGTTCAACGGCACAG-3’ | 140bp |
|  | R | 5’-GCCAGTAGACTCCACGACAT-3’ |  |
| Rat-PI3-Kp85 | F | 5’-AGGAGCGGTACAGCAAAGAC-3’ | 124bp |
|  | R | 5’-CTGCTGTCGATGATCTCGCT-3’ |  |
| Rat-IRS-1 | F | 5’-ATGTCGCCAGTGGGAGATT-3’ | 213bp |
|  | R | 5’-CTTCGGCAGTTGCGGTATA-3’ |  |
| Rat-Akt | F | 5’-TCTACGGTGCGGAGATTGT-3’ | 115bp |
|  | R | 5’-ATGTGCCCGTCCTTGTCCA-3’ |  |
